# The AMP-activated protein kinase beta 1 subunit modulates erythrocyte integrity

**DOI:** 10.1016/j.exphem.2016.09.006

**Published:** 2017-01

**Authors:** Emma L. Cambridge, Zoe McIntyre, Simon Clare, Mark J. Arends, David Goulding, Christopher Isherwood, Susana S. Caetano, Carmen Ballesteros Reviriego, Agnieszka Swiatkowska, Leanne Kane, Katherine Harcourt, The Sanger Mouse Genetics Project, David J. Adams, Jacqueline K. White, Anneliese O. Speak

**Affiliations:** a Wellcome Trust Sanger Institute, Wellcome Trust Genome Campus, Hinxton, Cambridgeshire, UK; b University of Edinburgh Division of Pathology, Centre for Comparative Pathology, Institute of Genetics & Molecular Medicine, Western General Hospital, Edinburgh, UK

## Abstract

Failure to maintain a normal in vivo erythrocyte half-life results in the development of hemolytic anemia. Half-life is affected by numerous factors, including energy balance, electrolyte gradients, reactive oxygen species, and membrane plasticity. The heterotrimeric AMP-activated protein kinase (AMPK) is an evolutionarily conserved serine/threonine kinase that acts as a critical regulator of cellular energy balance. Previous roles for the alpha 1 and gamma 1 subunits in the control of erythrocyte survival have been reported. In the work described here, we studied the role of the beta 1 subunit in erythrocytes and observed microcytic anemia with compensatory extramedullary hematopoiesis together with splenomegaly and increased osmotic resistance.

## Highlights

• *Prkab1*^*tm1b/tm1b*^ mice were generated and phenotyped.
• *Prkab1*^*tm1b/tm1b*^ mice presented with microcytic anemia.
• Erythrocytes lacking *Prkab1* have a variable morphologic appearance.
• *Prkab1*-deficient erythrocytes have increased osmotic resistance.
• The importance of the beta 1 isoform in erythrocyte integrity is discussed.

Erythrocytes are enucleated, terminally differentiated cells with a finite life span and an estimated turnover of 1% every day. To deal with stress, hemolysis, and/or hypoxia, the production of erythrocytes can be substantially modulated. In vivo control of erythrocyte survival is affected by many factors, including energy balance, maintenance of electrolyte gradients, and control of reactive oxygen species. Alterations to erythrocyte membrane deformability have a major role in regulating cellular function and intravascular survival, with reduced deformability resulting in splenic sequestration of abnormal cells, shortened half-life, and the clinical presentation of hemolytic anemia [1].

The evolutionary conserved serine/threonine kinase AMP-activated protein kinase (AMPK) is a critical regulator of energy balance 2, 3. AMPK is a heterotrimeric complex containing a catalytic alpha subunit paired with beta and gamma regulatory subunits. There are several isoforms for each subunit encoded by separate genes, two alpha (*Prkaa1* and *Prkaa2*), two beta (*Prkab1* and *Prkab2*), and three gamma (*Prkag1, Prkag2*, and *Prkag3*). *Prkaa1* and *Prkag1* can control oxidative stress, erythrocyte-intrinsic cellular metabolic stress, and membrane elasticity, making them critical regulators of erythrocyte integrity and life span 4, 5, 6, 7. However, the specific role of beta subunit isoforms in the context of erythrocyte development has not been studied.

Here we report that *Prkab1*-deficient mice present with splenomegaly, increased splenic iron deposits, microcytic anemia, compensatory extramedullary hematopoiesis, altered erythrocyte morphology, and increased erythrocyte osmotic resistance.

## Methods

### Mice

Generation of *Prkab1*^*tm1b(KOMP)Wtsi*^ (hereafter referred to as *Prkab1*^*tm1b*^) mice was performed using ES cell clone EPD0033_3_C09. Genotyping was carried out according to Ryder et al. [8] with cre conversion as reported [9]. All experiments were performed in accordance with the UK Home Office regulations and UK Animals (Scientific Procedures) Act 1986 and approved by the Wellcome Trust Sanger Institute animal welfare and ethical review body.

### Gene expression analysis

RNA was extracted from spleens using Purelink RNA mini kit (Ambion). Gene expression was assessed using FAM-conjugated TaqMan assays as listed in the Supplementary Methods (online only, available at www.exphem.org). Template RNA was added in duplex reactions in triplicate using *B2m* VIC primer limited probe (Mm00437762_m1) as the endogenous control using the EXPRESS One-Step Superscript qRT-PCR Kit (Thermo Scientific) and an Applied Biosystems 7900HT analyzer. Relative gene expression between endogenous control and target gene was analyzed using the ΔΔ*CT* method [10] with RQ manager (Life Technologies) applying automatic thresholds.

### Western blot analysis

Protein lysates were prepared from spleens, with protein quantification, electrophoresis, transfer, and antibody incubations performed according to standard protocols. Blots were visualized using horseradish peroxidase-conjugated secondary antibodies and ECL reagents, then imaged with a LAS 4000 (GE Healthcare). The primary antibodies used were AMPK beta 1 (1/1,000, No. 12063), AMPK beta 2 (1/1,000, No. 4148), AMPK pan alpha (all Cell Signalling Technology, 1/1,000, F6 No. 2793), and vinculin (Sigma, 1/5000, V284).

### Blood collection and analysis

Retro-orbital or tail vein blood was collected into EDTA-coated tubes for hematology or heparinized tubes for plasma preparation. Complete blood counts were determined using a Scil Vetabc system. Plasma was analyzed for bilirubin, iron, and ferritin using an Olympus AU400 analyzer (Beckman Coulter) with reagents supplied by Beckman Coulter or Randox. Erythropoietin was determined using a Meso Scale Discovery array.

### Histologic analysis

Spleen, liver, and leg bones were fixed in formalin and embedded in paraffin, and sections were stained with hematoxylin and eosin or Perls’ Prussian blue according to standard methods. These were assessed in a blinded manner for any pathologic abnormalities. Scanning electron microscopy (SEM) was performed as previously described [11] with erythrocytes adhered to poly-L-lysine-coated coverslips.

### Erythropoiesis analysis

Staining of single-cell suspensions of spleen, bone marrow, and whole blood with CD71, Ter119, CD45, Syto 16, and Sytox blue was performed as previously described [12] and analyzed on a BD LSRII instrument (full details in Supplementary Methods).

### In vivo clearance of erythrocytes

This was performed as described previously [4] with the exception that samples were labeled with either 10 μmol/L Vybrant CFDA (*Prkab1*^*+/+*^) or 1 μmol/L CellTracker Deep red (*Prkab1*^*tm1b/tm1b*^, both Molecular Probes). Erythrocytes were counted and adjusted to 2 × 10^6^ RBC/μL, and the two genotypes were pooled and injected via the tail vein into recipient mice (10 weeks old) to transfuse 2 × 10^8^ RBCs/genotype (full details in Supplementary Methods).

### Osmotic resistance assay

This was performed essentially as described [4] with hematocrit adjusted to 0.8% with 0.9% saline solution.

### Statistical analysis

All data was analyzed in Prism Version 6 (Graph Pad) and analyzed with an unpaired two-tailed Student *t* test, Mann–Whitney test or two-way analysis of variance as indicated in the figure legends.

## Results

**Supplementary Figure E1:**
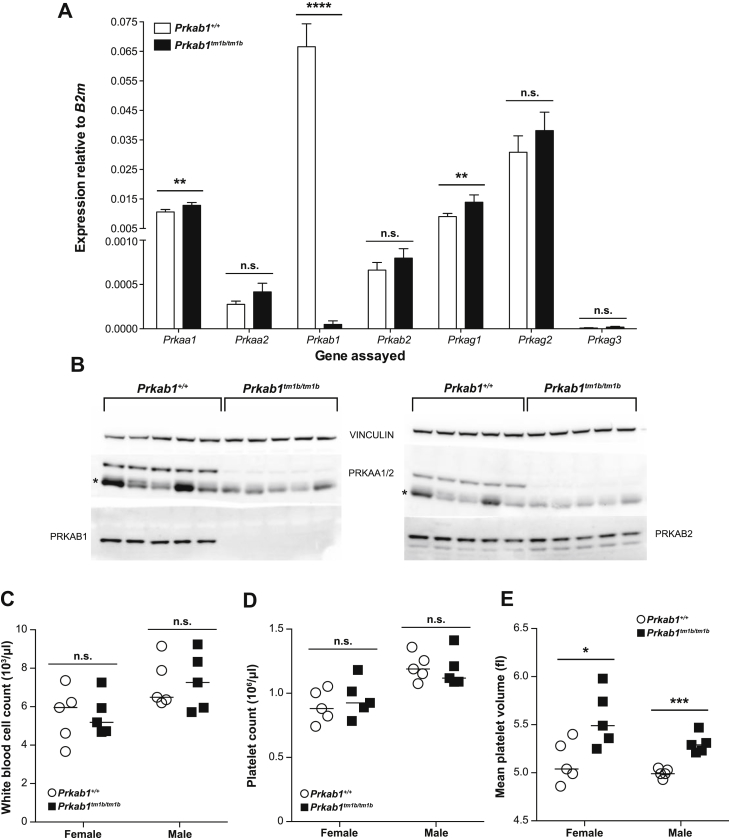
Molecular and phenotypic characterization of *Prkab1*-deficient mice. (**A**) Mean gene expression of AMPK subunits in *Prkab1*^*+/+*^ and *Prkab1*^*tm1b/tm1b*^ spleen RNA. n = 5 per genotype, with error bars representing standard errors of the mean. (**B**) Immunoblot analysis of AMPK subunits in *Prkab1*^*+/+*^ and *Prkab1*^*tm1b/tm1b*^ spleen protein lysates. *IgG heavy chain. (**C**) White blood cell count. (**D**) Mean platelet volume. (**E**) Platelet count of 16-week-old *Prkab1*^*+/+*^ and *Prkab1*^*tm1b/tm1b*^ mice. *p < 0.05, **p < 0.01, *** *p* < 0.001, and ****p < 0.0001. Student’s *t* test, hematology data are representative of three independent experiments. Each symbol represents an individual mouse with the line at the mean.

*Prkab1*^*tm1b/tm1b*^ mice exhibited greatly reduced expression of *Prkab1* that was accompanied by a significant (possibly compensatory) increase in *Prkaa1* and *Prkag1* (Supplementary Figure E1A, online only, available at www.exphem.org). This was confirmed by immunoblot analysis, which supports observations from *Prkag1* knockout mice [4] and another *Prkab1* knockout mouse line [13] that genetic deletion of one part of the AMPK heterotrimeric complex results in protein dysregulation of other parts of the complex, as there was no detectable alpha protein (pan-AMPK alpha antibody) in *Prkab1*^*tm1b/tm1b*^ spleen lysates (Supplementary Figure E1B).

**Figure 1:**
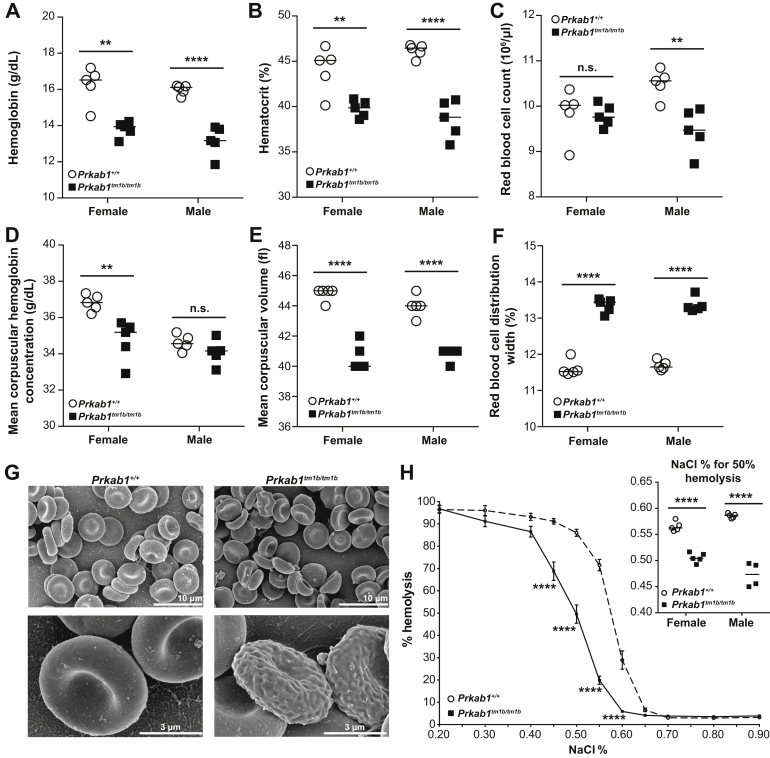
*Prkab1*-deficient mice present with anemia, erythrocyte morphologic abnormalities, and increased erythrocyte osmotic resistance. (**A**) Hemoglobin, (**B**) hematocrit, (**C**) red blood cell count, (**D**) mean corpuscular hemoglobin concentration, (**E**) mean corpuscular volume, and (**F**) red blood cell distribution width of 16-week-old *Prkab1*^*+/+*^ and *Prkab1*^*tm1b/tm1b*^ mice. **p < 0.01, and ****p < 0.0001, unpaired two-tailed Student t test. (**G**) Representative SEM images of erythrocytes from *Prkab1*^*+/+*^ and *Prkab1*^*tm1b/tm1b*^ mice. (**H**) Osmotic resistance of *Prkab1*^*+/+*^ and *Prkab1*^*tm1b/tm1b*^ erythrocytes (combined males and females). ****p < 0.0001 as determined by a repeated-measures two-way analysis of variance with Sidak's multiple comparison test adjusting for multiple testing, the insert is % of NaCl for 50% hemolysis of erythrocytes ****p < 0.0001, unpaired two-tailed Student *t* test. All data are representative of three independent experiments or two mice for SEM analysis. Each symbol represents an individual mouse with the line at the mean except for (**H**), where n = 10 for *Prkab1*^*+/+*^ and n = 9 for *Prkab1*^*tm1b/tm1b*^ with mean ± standard error of the mean.

**Supplementary Figure E2:**
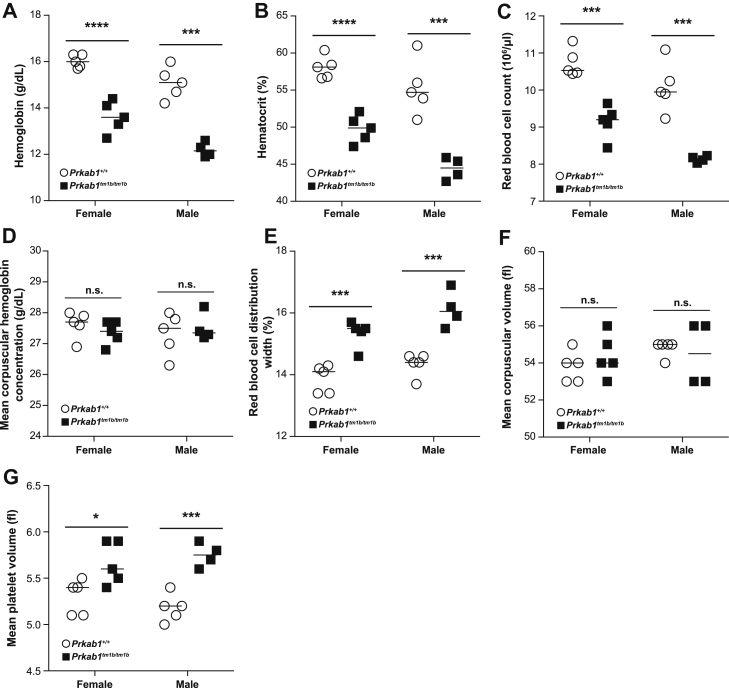
*Prkab1*^*tm1b/tm1b*^ mice have altered hematologic parameters at 4 weeks of age. (**A**) Hemoglobin, (**B**) hematocrit, (**C**) red blood cell count, (**D**) mean corpuscular hemoglobin concentration, (**E**) red blood cell distribution width, (**F**) mean corpuscular volume, and (**G**) mean platelet volume of 4-week-old *Prkab1*^*+/+*^ and *Prkab1*^*tm1b/tm1b*^ mice. *p < 0.05, ***p < 0.001, and ****p < 0.0001, unpaired two-tailedStudent *t* test. Data are representative of two independent experiments. Each symbol represents an individual mouse with the line at the mean.

At 16 weeks of age, *Prkab1*^*tm1b/tm1b*^ mice had significantly reduced hemoglobin (Fig. 1A) and hematocrit (Fig. 1B). Reductions in erythrocyte number (Fig. 1C) and mean corpuscular hemoglobin concentration (Fig. 1D) were observed only in a sex-specific manner; however, erythrocytes in *Prkab1*^*tm1b/tm1b*^ mice were significantly smaller (Fig. 1E), with an increased red blood cell distribution width (Fig. 1F) in both sexes. These altered erythrocyte indices indicate a microcytic anemia with anisocytosis, similar to that reported in mice deficient in *Prkaa1* or *Prkag1* 4, 5, 6, 7. The leukocyte lineage was unaffected by deletion of *Prkab1* (Supplementary Figure E1C), and there were no differences in the circulating platelet count (Supplementary Figure E1D). However, there was an increase in the size of the platelets in both sexes (Supplementary Figure E1E). At 4 and 6 weeks of age, the anemia was normocytic (Supplementary Figure E2A–G, online only, available at www.exphem.org; data not shown).

Scanning electron microscopy confirmed anisocytosis; erythrocytes from *Prkab1*^*tm1b/tm1b*^ mice varied in appearance, with features of acanthocytes, schistocytes, stomatocytes, and echinocytes (Fig. 1G). We then determined osmotic resistance; *Prkab1*-deficient erythrocytes had a left-shifted curve indicative of increased osmotic resistance (Fig. 1H), in agreement with the previously observed findings in *Prkaa1-*and *Prkag1-*deficient mice 4, 5, 6, 7.

**Figure 2:**
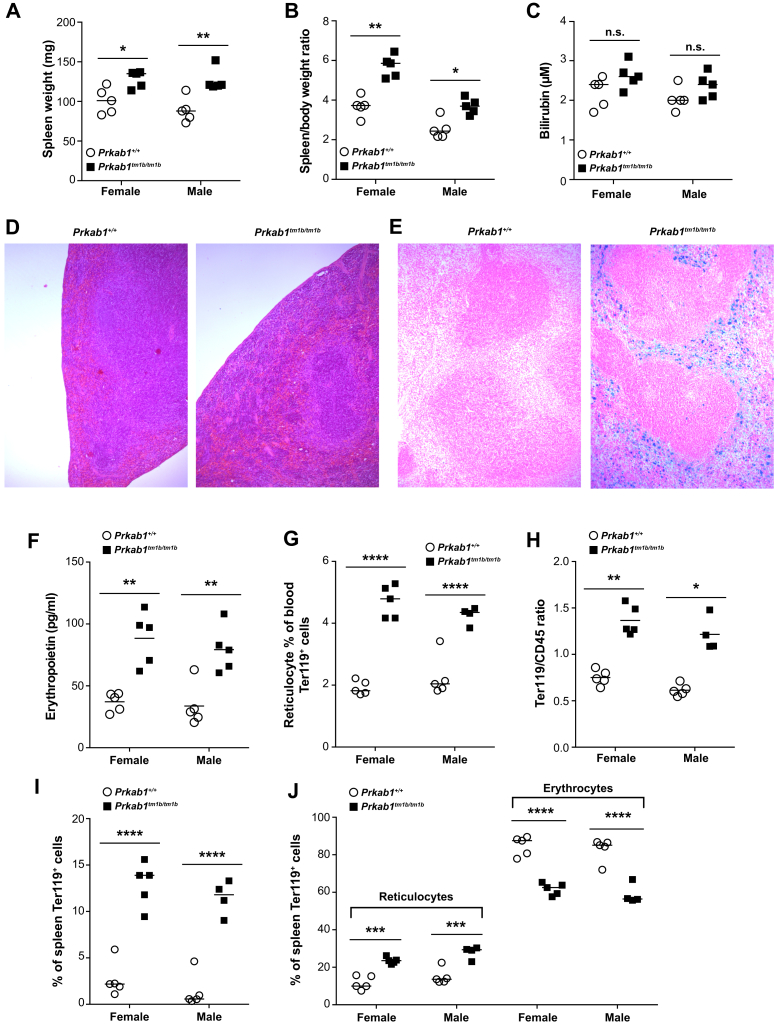
*Prkab1*-deficient mice have splenomegaly, extramedullary hematopoiesis, and splenic iron deposits. (**A**) Spleen weight. (**B**) Spleen/body weight ratio (mg/g). (**C**) Plasma bilirubin concentration. (**D**) Hematoxylin and eosin-stained sections of spleen (100× magnification). (**E**) Perls’-stained sections of spleen (100× magnification). (**F**) Plasma erythropoietin. (**G**) Percentage of circulating reticulocytes. (**H**) Splenic erythroid (Ter119)/leukocyte (CD45) ratio. (**I**) Percentage of splenic erythroblasts. (**J**) Percentages of splenic reticulocytes and erythrocytes. For all, *p < 0.05, **p < 0.01, ***p < 0.001, and ****p < 0.0001, unpaired two-tailed Student *t* test except for spleen/body weight ratio and Ter119/CD45 ratio, which were analyzed with a Mann–Whitney test. All data are representative of three independent experiments or four mice for histology analysis; each symbol represents an individual mouse with the line at the mean.

**Supplementary Figure E3:**
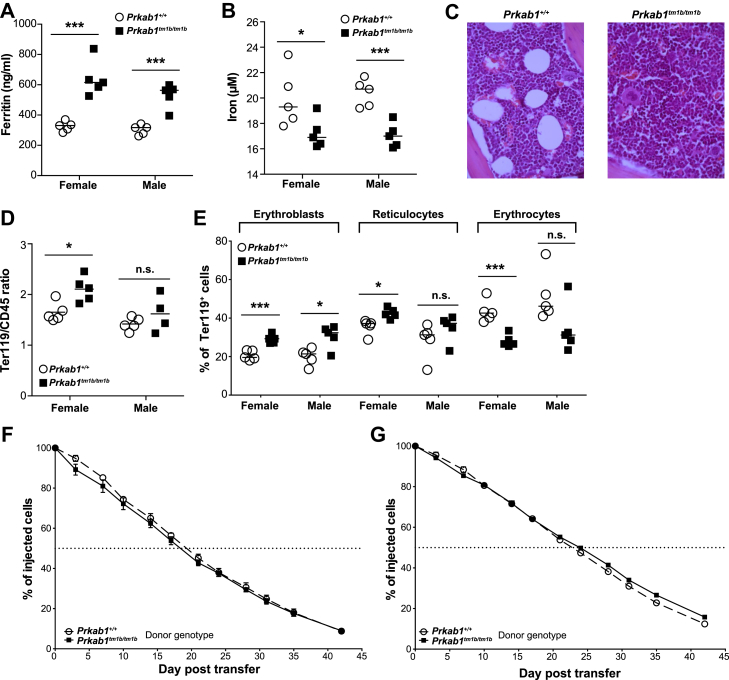
Characterofization of circulating iron, bone marrow erythropoiesis, and erythrocyte half-life of *Prkab1*-deficient mice. (**A**) Plasma ferritin concentration. (**B**) Plasma iron concentration. (**C**) Representative H&E stained bone marrow sections from *Prkab1*^*+/+*^ and *Prkab1*^*tm1b/tm1b*^ mice (400× magnification). (**D**) Erythroid (Ter119)/Leukocyte (CD45) ratio of bone marrow from *Prkab1*^*+/+*^ and *Prkab1*^*tm1b/tm1b*^ mice. (**E**) Characterization of erythropoiesis in the bone marrow of *Prkab1*^*+/+*^ and *Prkab1*^*tm1b/tm1b*^ mice. In vivo half-life of erythrocytes transferred into *Prkab1*^*+/+*^ (**F**) or *Prkab1*^*tm1b/tm1b*^ (**G**) mice. For all, *p < 0.05, ***p < 0.001, and ****p < 0.0001, unpaired two-tailed Student *t* test, except for Ter119/CD45 ratio, which was analyzed with a Mann–Whitney test. Data are representative of two independent experiments or four mice for histology analysis. Each symbol represents an individual mouse with the line at the mean, except for (**F**) and (**G**), where n = 5 with mean ± standard error of the mean.

At necropsy, *Prkab1*^*tm1b/tm1b*^ mice presented with splenomegaly (Fig. 2A and B), although not to the same degree as *Prkag1*^*−/−*^ and *Prkaa1*^*−/−*^ mice 4, 5, 6, 7. We determined the level of total bilirubin in the plasma, an indicator of erythrocyte destruction, and although increased in *Prkab1*^*tm1b/tm1b*^ mice, this did not reach significance in any of the cohorts tested (Fig. 2C). *Prkab1*^*tm1b/tm1b*^ spleens exhibited an expansion of the peripheral red pulp caused by increased extramedullary hematopoiesis and increased red cell breakdown with hemosiderin in the red pulp (Fig. 2D). Hemolytic anemia often results in changes in tissue iron deposits, and we found a significant increase in splenic iron deposits in *Prkab1*^*tm1b/tm1b*^ mice (Fig. 2E), with a concomitant increase in circulating levels of ferritin (Supplementary Figure E3A, online only, available at www.exphem.org) and decrease in iron concentration (Supplementary Figure E3B). Circulating erythropoietin was significantly increased in *Prkab1*^*tm1b/tm1b*^ mice (Fig. 2F), as was the percentage of reticulocytes (Fig. 2G).

There was an increase in the ratio of Ter119^+^ to CD45^+^ cells in the spleen (Fig. 2H), as well as increase, in percentages of erythroblasts (Fig. 2I) and reticulocytes, with a concomitant decrease in mature erythrocytes (Fig. 2J). The bone marrow exhibited a reduction in adipocytes of the marrow stroma and a mild hematopoietic hyperplasia with a mild increase in erythroid subsets (Supplementary Figure E3C–E). These observations would suggest a reactive increase in erythroid hematopoiesis in both bone marrow and spleen in response to the observed hemolytic anemia. A similar hemolytic anemia with compensatory extramedullary hematopoiesis has been observed in *Prkaa1-*and *Prkag1-*deficient mice 4, 5, 6, 7.

Previous studies on *Prkag1*^*−/−*^ and *Prkaa1*^*−/−*^ mice have found that deficiency in either gene results in a decreased half-life in vivo 4, 5, 7. Via adoptive transfer of fluorescence- labeled erythrocytes, we observed no difference in the half-life of *Prkab1*^*tm1b/tm1b*^ erythrocytes when transferred into wild-type mice, compared with the co-transferred wild-type erythrocytes (Supplementary Figure E3F), or when transferred into *Prkab1*^*tm1b/tm1b*^ mice (Supplementary Figure E3G). However, we cannot rule out the possibility that the method employed skews the analysis if the ex vivo fluorescence labeling preferentially occurs in “normal” erythrocytes given the heterogeneous morphologic alterations to the erythrocytes in *Prkab1*^*tm1b/tm1b*^ mice.

## Discussion

In summary we report a key role for the AMPK beta 1 subunit in erythrocyte development similar to that observed for alpha 1 and gamma 1 subunits. Deletion of *Prkab1* resulted in regenerative hemolytic anemia, splenomegaly, and splenic iron deposition with enhanced erythropoiesis in the spleen and, to a lesser extent, bone marrow. Erythrocytes from deficient mice presented with multiple morphologic alterations and increased osmotic resistance.

## Supplementary data

### Supplementary methods

#### Gene expression analysis

The FAM conjugated TaqMan Gene Expression Assays used were as follows: *Prkaa1* (Mm01296700_m1), *Prkaa2* (Mm01264789_m1), *Prkab1* (Mm01201921_m1), *Prkab2* (Mm01257133_m1), *Prkag1* (Mm00450298_g1), *Prkag2* (Mm00513977_m1), *Prkag3* (Mm00463997_m1).

#### Western blot analysis

Spleen samples were homogenized in IP lysis buffer (Thermo Fisher) supplemented with protease and phosphatase inhibitors (cOmplete and PhosSTOP both Roche) using M tubes and an OctoMACS (Miltenyi Biotec) with program protein_01. Samples were left on ice for 15 min and then cleared by centrifugation for 10 min at 20,000*g* at 4°C. The protein concentration of the supernatant was determined using the BCA kit from Pierce according to the manufacturer's instructions. Protein samples (15 μg/lane) were prepared for electrophoresis on 4–12% 1.0 mm NuPage Bis–Tris gels with 4× LDS buffer and 10× sample reducing agent (all from Invitrogen). Novex sharp prestained molecular weight ladders were also applied to the gel, and MOPS running buffer (Invitrogen) was used for protein separation. Proteins were transferred to PVDF membrane using wet transfer in the XCell II blot module. Once transferred, membranes were blocked with 5% nonfat milk powder in Tris-buffered saline with 0.1% Tween 20 (TBS-T) for 1 hour with rocking at room temperature. Blots were then cut to allow them to be separately probed with primary antibodies using the molecular weight markers as a guide. Primary antibodies were all prepared in 2.5% nonfat milk in TBS-T and incubated overnight at 4°C with rocking. Blots were washed before incubation in horseradish peroxidase-conjugated secondary antibodies for 90 min at room temperature (anti-rabbit or anti-mouse HRP [Abcam] 1/5,000 dilution in 2.5% nonfat milk in TBS-T). After being washed, blots were visualized using an enhanced chemiluminescence spray (Advansta, National Diagnostics) and imaged in an LAS 4000 (GE Healthcare, Chalfont). Primary antibodies were used against AMPK beta 1 (Cell Signalling Technology, 1/1000, No. 12063), AMPK beta 2 (Cell Signalling Technology, 1/1,000, No. 4148), and AMPK pan alpha (Cell Signalling Technology, 1/1,000, F6 No. 2793), and vinculin (Sigma, 1/5000, V284) acted as a loading control.

#### Erythropoiesis analysis

Single-cell suspensions were prepared from spleen and bone marrow using D-PBS according to standard methods. Cells from spleen and bone marrow plus blood were stained for 30 min at 4°C with Ter119-APC (0.33 μg/mL, TER-119, Biolegend), CD71-PE-Cy7 (0.1 μg/mL, RI7217, Biolegend), and CD45-Alexa Fluor 700 (0.833 μg/mL, 30-F11, Biolegend) antibodies after blocking with 1 μg Mouse FC block (2.4G2, BD Biosciences) for 10 min. After being washed, the samples were incubated in 0.5 μmol/L Syto 16 and 1 μmol/L Sytox Blue (both Invitrogen) for 15 min at room temperature, prepared in FACS buffer (Dulbecco's phosphate-buffered saline without calcium or magnesium [D-PBS, Gibco], supplemented with 1% bovine serum albumin [Sigma]). Samples were washed prior to acquisition on a BD LSRII instrument. Dead cells were excluded (Sytox Blue^high^) and singlets by FSC-A versus FSC-H and SSC-H versus SSC-W. Erythroid cells were identified as Ter119^+^ and leukocytes as CD45^+^, with erythroblasts CD71^+^ Syto 16^high^, reticulocytes CD71^high^ Syto 16^low^, and mature erythrocytes as CD71^–^ Syto 16^neg^.

#### In vivo clearance of erythrocytes

Blood (700 μL) was collected under terminal anesthesia from the retro-orbital sinus into EDTA-coated tubes and was washed twice with 14 mL of D-PBS to remove all serum proteins that would quench the fluorescence labeling. Blood from two mice of the same genotype were pooled and labeled with either 10 μmol/L Vybrant CFDA (wild type) or 1 μmol/L CellTracker Deep Red (mutants) for 30 min at 37°C with constant gentle mixing by rotation. The reaction was quenched by the addition of 10 vol of D-PBS containing 5% fetal bovine serum (Sigma). Erythrocytes were pelleted and washed twice with D-PBS prior to counting using a Scil Vetabc system. Erythrocyte concentrations of each genotype sample were adjusted with D-PBS to 2 × 10^6^ RBC/μL. The two genotypes were then pooled, and 200 μL was injected into C57BL/6N recipient mice (10 weeks old) to transfuse 2 × 10^8^ RBCs/genotype. Blood samples (2 μL) were collected at the indicated time points and placed in 3 mL of flow cytometry buffer (D-PBS with 2 mmol/L EDTA and 0.5%). The samples were mixed and acquired on a BD LSRII instrument with CFDA detected in the FITC channel (excitation: 488 nm, emission: 530/30) and CellTracker Deep Red in the APC channel (excitation: 633, emission: 660/20). Gates were set around the CFDA or CellTracker Deep Red single positive erythrocytes, and the percentage of total erythrocytes was determined. A total of 5,000 labeled erythrocytes were acquired, and application settings in BD FACSDiva software were used to standardize the instrument voltage settings over the experiment duration. The percentage of fluorescent erythrocytes was calculated as the percentage of the total labeled fraction determined from the blood collected 60 min after injection.

#### Osmotic resistance assay

Blood was collected as described above, and hematocrit levels were determined using a Vetabc analyzer. The hematocrit of the samples was adjusted to 0.8% with 0.9% saline solution. This 0.8% hematocrit solution was added to 12 wells of a 96-well V-bottom plate (per mouse), and the erythrocytes were pelleted (400*g*, 3 min). The supernatant was discarded, and the erythrocytes were resuspended in 200 μL of saline solutions or water as indicated in the figure. The samples were left for 1 hour at room temperature, and erythrocytes were pelleted. The supernatant (75 μL) was transferred to a flat-bottom plate, and the absorbance at 405 nm was measured. The percentage hemolysis was determined by normalizing the reading of erythrocytes in water to the saline solutions.
